# Spatial and temporal variability of metal(loid)s concentration as well as simultaneous determination of five arsenic and antimony species using HPLC-ICP-MS technique in the study of water and bottom sediments of the shallow, lowland, dam reservoir in Poland

**DOI:** 10.1007/s11356-020-07758-9

**Published:** 2020-01-28

**Authors:** Magdalena Jabłońska-Czapla, Katarzyna Grygoyć

**Affiliations:** Institute of Environmental Engineering, Polish Academy of Sciences, 34 M. Skłodowskiej-Curie Street, 41-819 Zabrze, Poland

**Keywords:** Arsenic speciation, Antimony speciation, Dam reservoir, Bottom sediment, Heavy metals, PLI, HPLC-ICP-MS, BCR

## Abstract

The optimization of new methodology for simultaneous determination of arsenic [As(III), As(V)] and antimony [Sb(III), Sb(V), SbMe_3_] species using high-performance liquid chromatography (HPLC) coupled with inductively coupled plasma mass spectrometry (ICP-MS) in water and bottom sediment samples collected from the dam Kozłowa Góra Reservoir (Poland) was studied. Samples were collected monthly from May to September 2018 in four-point (water) and fifth-point (sediment) transects. The contents of Mn, Co, Ni, Cu, Zn, As, Cr, Rb, Sr, Cd, Sb, Ba, Tl, Pb, and Sb were studied in water and bottom sediments using ICP-MS techniques. Additionally, arsenic and antimony fractions were determined in sediments with the BCR method. Pollution Load Index (PLI), Geoaccumulation Index (I_geo_), LAWA classification, and Sb/As ratio indicated the presence of extreme sediment pollution for Zn, Cd, Pb, and Cr from anthropogenic sources. Research has shown that the easy-leached bottom sediment fraction contained in most cases more As(V) and Sb(V). But often Sb(V) concentration was equal as Sb(III), which can be released into the pelagic zone under favorable conditions. Even though As(V) and Sb(V) prevail in the reservoir bottom sediments, they can be transformed into As(III) and Sb(III) as a result of drastic changes in pH or redox potential. The Kozłowa Góra sediments are heavily polluted with Pb, Zn, Cd, and As, Cu, and Ni. The highest concentrations of the heavy metals were recorded in the middle of the tank and there was a small spatial variability. The migration of metals along the reservoir transect was closely related to its morphometry.

## Introduction

The Upper Silesia area lacks natural lakes. All the reservoirs in the region are anthropogenic. Nonetheless, they constitute valuable environment elements in terms of the environment itself, economy, and landscape. Our previous studies (Jabłońska-Czapla et al. 2014, 2015) have shown that dam reservoirs differ considerably due to metal and metalloid contents, including ionic arsenic and antimony forms. There are many reasons for differences in arsenic and antimony species content in the water and sediments of the reservoir. One of them is the relationship of the reservoir function: the Goczałkowice drinking water reservoir (Jabłońska et al. 2012), the Rybnik Reservoir for cooling the water in the Rybnik Power Plant (Jabłońska-Czapla et al. 2015), or a tank used mainly for recreation (Michalski et al. 2016, 2019). Each of these tanks differs in morphometry, flow, physicochemical conditions prevailing in the water and bottom sediments. In the case of water reservoirs, its location is extremely important. In the Upper Silesia agglomeration, the influence of the mining and zinc and lead ores industry is the largest. Eutrophication plays a major role in the evolution of water bodies, in which intensification is observed primarily in areas with pronounced anthropogenic influences due to the increased supply of organic and mineral matter, leading in consequence to shallowing and then disappearing of the reservoir. In the Upper Silesia region, the course of eutrophication processes is considered extremely dynamic, which is favored by sewage emission, agricultural intensification, forest cutting, and atmospheric pollution (Rogula-Kozłowska et al. 2013, 2015). The universality of the eutrophication phenomenon of stagnant water means the modification of many natural processes (thermal, oxygen, oxidation and reduction, sedimentation), and also results in difficulties in the use of reservoirs and the use of their margins (Granero et al. 2004). Bottom sediments of water reservoirs are an important element of aquatic ecosystems, taking an active part in the geochemical cycle of elements and organic matter. Trace elements including Mn, Co, Ni, Cu, Zn, As, Cr, Rb, Sr, Cd, Ba, Tl, Pb, Sb get into surface waters, and then, they are accumulated in bottom sediments, through various routes. It is mainly the discharge of municipal and industrial sewage to water reservoirs, surface runoff from arable fields (agricultural activity), dust fall and transport (Bijendra and Anshumali 2019). Contamination of bottom sediment anthropogenic dam reservoir is a global problem and is being investigated by researchers in many countries (Akin and Kirmizigul 2017; Goher et al. 2014; Hahn et al. 2018; Karadede and Unlu 2000). One of the fundamental characteristic of metals is their lack of biodegradability. The possible increase acidification of the some reservoir environment water bodies is a specific threat, which can be equated with the uncontrolled increase in the mobility of metals, currently accumulated in bottom sediments and their transfer to the water. Exceeding the trace element concentration in the bottom sediments, in comparison to their level usually found in sedimentary rocks, is an essential indicator of anthropopressure. This is particularly clear in relation to the content of such elements as zinc, cadmium, and lead, whereas the cadmium concentration scale is many times greater than the natural bottom sediment content (0.05–0.35 mg/kg) of some reservoirs.

Migration of As and Sb ions from reservoir bottom sediments to the water and vice versa is a complex process, and understanding the content of particular ionic forms of selected elements is very important, in the context of understanding the changes taking place in the water reservoir ecosystems (Jachniak 2011; Rzętała 2008). This is particularly important in the case of water reservoirs performing the water supply function and used for recreational purposes. Toxicological properties of elements such as arsenic and antimony depend on the degree of oxidation on which they occur and are strongly different. It turns out that the determination of the total element content is insufficient to determine its biological and chemical properties, because the elements in the environment occur at various oxidation states, such as, e.g., Sb(III)/Sb(V) or As(III)/As(V) and in various combinations—chemical compounds. The most harmful for living organisms are the inorganic arsenic forms. The Sb(III) compounds are about 10 times more toxic than Sb(V) ones (Cornelis et al. 2005; Filella et al. 2002; Garboś et al. 2000; Jabłońska-Czapla 2015; Jabłońska-Czapla and Szopa 2016; Leonard and Gerber 1996; Marcellino et al. 2008; Semczuk 1990; Skorek et al. 2012). Antimony can be converted into methyl derivatives under bacteria influence (Filella 2010; Jenkins et al. 1998). There is a lack of information on methyl antimony derivatives in the environment (Yang and He 2015a, 2015b). SbMe_3_ is characterized by lower toxicity as inorganic antimony forms; however, as previous studies have shown, it also has genotoxic effects (Dopp et al. 2006).

Determining the heavy metal water pollution level at a satisfactory level of accuracy was not always possible. Only some instrumental analysis techniques allow the determination of the metal content in water with proper accuracy. The use of spectroscopic methods such as AAS or ICP-MS (Skorek et al. 2012) allowed for determination of the total metals and metalloid content in the water at low concentration levels. Methods permitting the speciation form analysis of elements are hyphenated techniques (Komorowicz and Barałkiewicz 2011; Michalski et al. 2011, 2012, 2013a, b; Michalski and Szopa 2017; Szopa and Michalski 2015). One of these techniques is liquid chromatography coupled with inductively coupled plasma mass spectrometry HPLC-ICP-MS (Jabłońska et al. 2012; Jabłońska-Czapla 2015; Jabłońska-Czapla et al. 2014, 2015; Jabłońska-Czapla and Szopa 2016).

The main objectives of the study were (1) developing and validating the methodology of simultaneous determination of five species: As(III), As(V), Sb(III), Sb(V), and SbMe_3_ in water and bottom sediments using HPLC-ICP-MS hyphenated technique; (2) determination of the temporal and spatial distribution of Mn, Co, Ni, Cu, Zn, As, Cr, Rb, Sr, Cd, Ba, Tl, Pb, Sb, as well as arsenic and antimony species and enabled the assessment of hazards associated with the use of this reservoir in both recreation (bathing) and as a source of drinking water; (3) determination of the metal(loid)s mobility (including arsenic and antimony species) in surface water, bottom water, and bottom sediments of the Kozłowa Góra Reservoir using sequential chemical extraction procedure proposed by BCR (the Institute for Reference Materials and Measurements) (Fadiran et al. 2014); (4) assessment of the ecological risks arising from metal(loid)s contamination though sediment quality and determination whether deposited bottom sediments of the reservoir are a potential ecological threat, which under favorable redox conditions can release into the water depth a lot of toxic As(III) and Sb(III) species; (5) description of the extant of metal(loid)s pollution using Pollution Load Index (PLI) (Tomlinson et al. 1980), Geoaccumulation Index (Igeo) (Müller 1969), LAWA classification (LAWA 1998), and Sb/As ratio (Bi et al. 2011; Fu et al. 2011; Sharifi et al. 2016).

## Materials and methods

### Research object

The object of the study was lowland, shallow, water supply, Kozłowa Góra dam reservoir, in which as a result of limestone ecosystem enrichment in biogenic substances, there was an increase in biomass of phytoplankton organisms. The increase in the amount of mineralized and deposited bottom sediment in the reservoir was the consequence of biomass growth in the reservoir. The Kozłowa Góra Reservoir (also known as the Świerklaniec Reservoir) is located north of the Upper Silesia industrial agglomeration and southeast of the extensive Lubliniecko-Tarnogórskie forest complex (south Poland). The reservoir is located far from the Odra and Vistula valleys. It was created by damming up the Brynica river (Jaguś and Rzętała 2003). In the vicinity of the reservoir, a water treatment station was established. Physicochemical properties of the Kozłowa Góra dam reservoir (agricultural and forest catchment) are the result of the impact of indigenous waters with different levels of pollution. The Kozłowa Góra Reservoir is largely eutrophicated; to a limited extent, it is used for tourism and recreation.

### Sampling and sample preparation

**Fig. 1:**
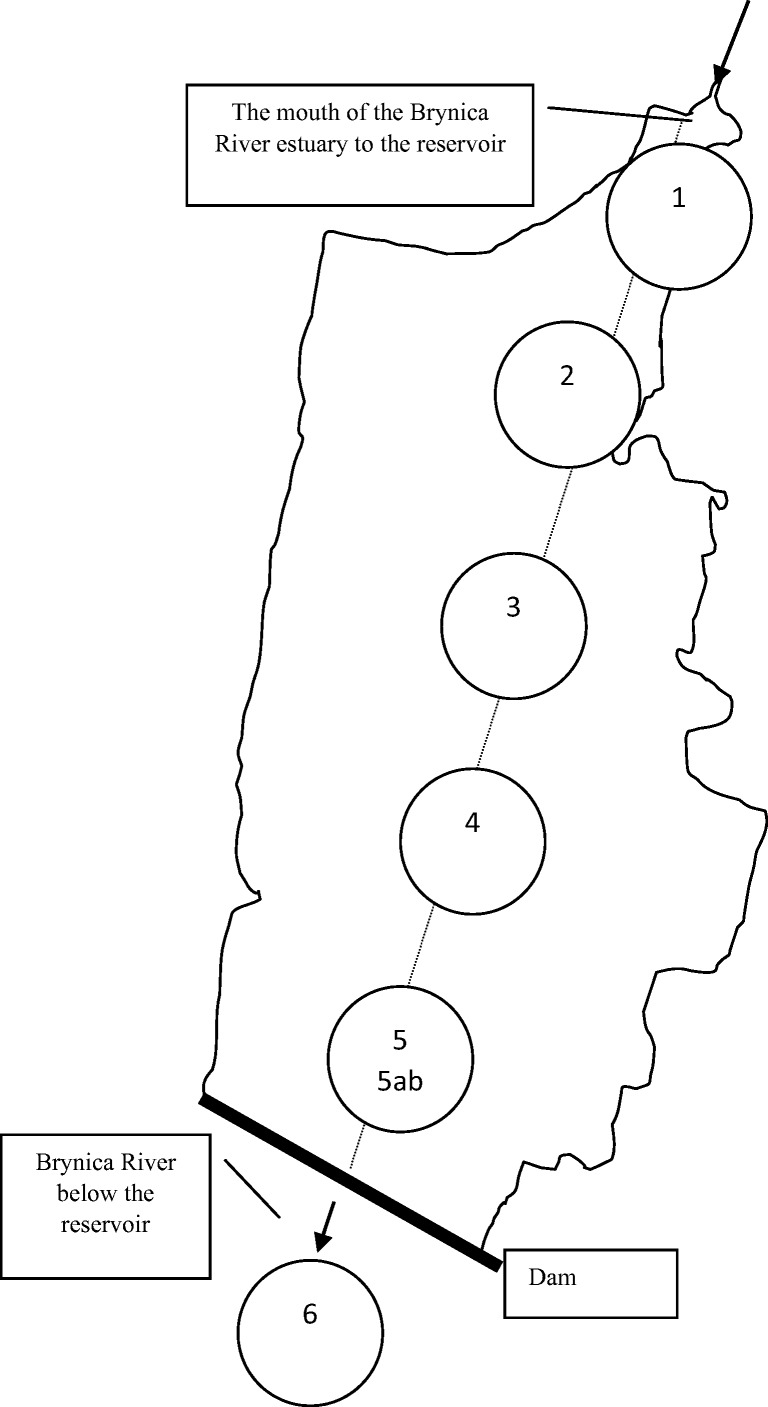
Water and bottom sediment sampling point of the Kozłowa Góra Reservoir. Water sampling points: 1–the inflow area of the Brynica River to the reservoir, 5a–the dam zone, surface water, 5b–the dam zone, bottom water, 6–Brynica River–outflow, below the dam. Bottom sediment sampling points: 1–5

The water and bottom sediment samples were collected monthly from May to September 2018. Figure 1 shows the locations of sampling points. The water of the Kozłowa Góra Reservoir was collected at three sampling points: in the inflow area of the Brynica River to the reservoir, in the dam zone (bottom and surface water), and in the water outflow area from the reservoir. The bottom sediments were collected at five points located in the longitudinal axis of the reservoir transect, with a Birge-Eckmann sampler from the layer with thickness of 0–5 cm. The bottom water was sampled with Ruttner water sampler.

The water samples were collected into 1000-mL HDPE containers, in this way to avoid unnecessary aeration. Immediately after transporting to the laboratory and determining basic physicochemical parameters (pH, conductivity, redox potential), the samples were divided into two parts. One of them was acidified by spectrally pure nitric acid (Merck, Germany) and after filtration through a PES syringe filter with pore size of 0.22 μm, designed to determine the total content of Mn, Co, Ni, Cu, Zn, As, Sb, Rb, Sr, Ag, Cd, Ba, Tl, Pb, Cr using ICP-MS technique. The second part of the laboratory water sample was placed in a HDPE container and stored at − 22 °C until analysis (not longer than a month). The samples before freezing were preserved by adding 20 μl of a saturated Na_2_EDTA solution to the 50 mL of water.

The bottom sediment samples after transporting to the laboratory and determining basic physicochemical parameters were stored at − 22 °C temperature until further analysis. The bottom sediment samples for the total metals and metalloid content were air-dried, pulverized, and sieved through a 2-mm sieve. Prepared samples were then digested in an Anton Paar Microwave 3000 oven (1200 W, 35 min). In the digestion spectral purity, 6 mL HCl, 2 mL HNO_3_, and 2 mL HF were used.

Bottom sediment samples for speciation analysis at the first stage of preparation were centrifuged, air dried, ground, and sieved through a 2-mm sieve. Determination of the total content of metal species in bottom sediments is practically impossible due to the drastic conditions that are necessary to release them (concentrated acids HNO_3_, HF, or perhydrol). The environment of concentrated acids, especially oxidizing ones, has an oxidizing effect on the species of analytes with lower oxidation states. Metal species associated with the lowest fractions are characterized by strong demobilization; hence, they are of little importance from a biological point of view. Therefore, it is important to quantify only metal species in the easily leached fractions of bottom sediments.

Each sediment sample was extracted in order to leach easy-leached As and Sb fractions from the sample. From the biological viewpoint, they have major significance. Extraction with a phosphate buffer was the most optimal for As. The 1-g bottom sediment samples were shaking in a shaker (165 rotations/min) for 2 h, at room temperature with 10 mL of the phosphate buffer (5 mM Na_2_HPO_4_ and 50 mM KH_2_PO_4_ pH = 6.0 ± 0.2). Unfortunately, antimony extraction with phosphate buffer causes high Sb background, and it was observed that Sb(III) oxidized to Sb(V) under these conditions and reagents. The best results were achieved when the antimony species were extracted with a weak Na_2_EDTA solution. Additionally, it stabilized and preserved the sample. The 1-g bottom sediment samples were shaking in a shaker (165 rotations/min) for 2 h, at room temperature with 10 mL of 20 mM Na_2_EDTA added for the extraction of the antimony ionic forms. After the obtained eluate was filtered through a syringe filter with pore size of 0.22 μm, the arsenic and antimony species were quantitatively determined for each analyte separately.

### Reagents

The following substances were used for analysis: disodium hydrogen arsenate heptahydrate (98.0%, Sigma-Aldrich, Germany), sodium arsenite (99.0%, Sigma-Aldrich, Germany), potassium hexahydroxoantimonate(V) (for analysis Emsure®, Sigma-Aldrich, Germany), trimethylantimony(V) bromide (98%, Sigma-Aldrich, Germany), antimony(III) oxide (99%, Sigma-Aldrich, Germany). To extract As and Sb species from the bottom sediments, the following substances were used: disodium ethylenediaminetetraacetate (Na_2_EDTA) (99.0–101%, Sigma-Aldrich, Germany), analytically pure dihydrogen potassium phosphate (KH_2_PO_4_) (POCH, Poland), analytically pure di-sodium hydrogen phosphate (Na_2_HPO_4_) (POCH, Poland). Phthalic acid (POCH, Poland) and Na_2_EDTA (99.0–101%, Sigma-Aldrich, Germany) were used as eluents in speciation analysis. To digest the bottom sediments, suprapur HCl (30% Merck, Germany), ultrapure HNO_3_ (65% Merck, Germany), and suprapur HF (40% Merck, Germany) were used. Multielemental standards no. XXI and VI (Merck, Germany) and Sb standard (Merck, Germany) were used during determination of total Mn, Co, Ni, Cu, Zn, As, Cr, Rb, Sr, Cd, Ba, Tl, Pb, Sb content for ICP-MS. Solutions made from salt were employed for calibration during quantitative analysis of As and Sb speciation forms. All solutions and standards were prepared with Milli-Q-Gradient ultrapure deionized water (Millipore, Merck), whose electrolytic conductivity was < 0.05 μS/cm.

### Apparatus

The Elan 6100 DRC-e spectrometer (Perkin Elmer) was used for quantitative analysis of total As, Sb, and other elements in the water and bottom sediments (digest and extracts). The apparatus was equipped with a standard ICP quartz torch, cross-flow nebulizer, and nickel cones. Samples and standards were delivered with a peristaltic pump. The spectrometer was optimized daily with a 10-μg/L solution (Mg, Cu, Rh, Cd, In, Ba, Ce, Pb, U) in 1% HNO_3_ Elan 6100 Setup/Stab./Masscal. Solution (Perkin-Elmer). Concentrations of ^55^Mn, ^59^Co, ^60^Ni, ^65^Cu, ^66^Zn, ^75^As, ^53^Cr, ^85^Rb, ^88^Sr, ^114^Cd, ^123^Sb, ^138^Ba, ^205^Tl, ^208^Pb were measured with the internal ^103^Rh standard. The measurements of As and Sb species in the water and bottom sediment samples were performed with the HPLC-ICP-MS system. To separate analytes, a speciation apparatus set was applied. It consisted of an HPLC chromatograph (Perkin Elmer), Series 200LC Peltier oven, Series 200 autosampler, and series 200LC gradient pump. As(III), As(V), Sb(III), Sb(V), and SbMe_3_ ions were separated with the Dionex IonPac AS7 (200 mm × 4 mm, particle size 10 μm, Dionex). Selected separation parameters are presented in Table 1.

**Table 1:** Conditions of chromatographic separation ions: As(III), As(V), Sb(III), Sb(V), SbMe_3_

Parameter	Value
Separation column	Dionex IonPac AS 7 (10 μm, 250 × 4 mm)
Temperature	30 °C
Mobile phase	1.5 mmol/L phthalic acid + 10 mmol/L Na_2_EDTA (pH = 4.0)
Elution program	6 min
Flow rate during the analysis [mL/min]	0.7
Volume of sample [μL]	150

### Calibration

**Fig. 2:**
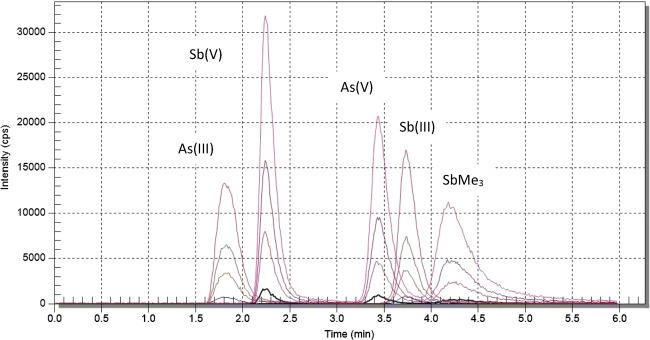
Superimposed chromatograms obtained after analyzing standard solutions of As(III), As(V), Sb(III), Sb(V), and SbMe_3_ with concentrations of 1 μg/L, 5 μg/L, 10 μg/L, 20 μg/L, respectively

**Fig. 3:**
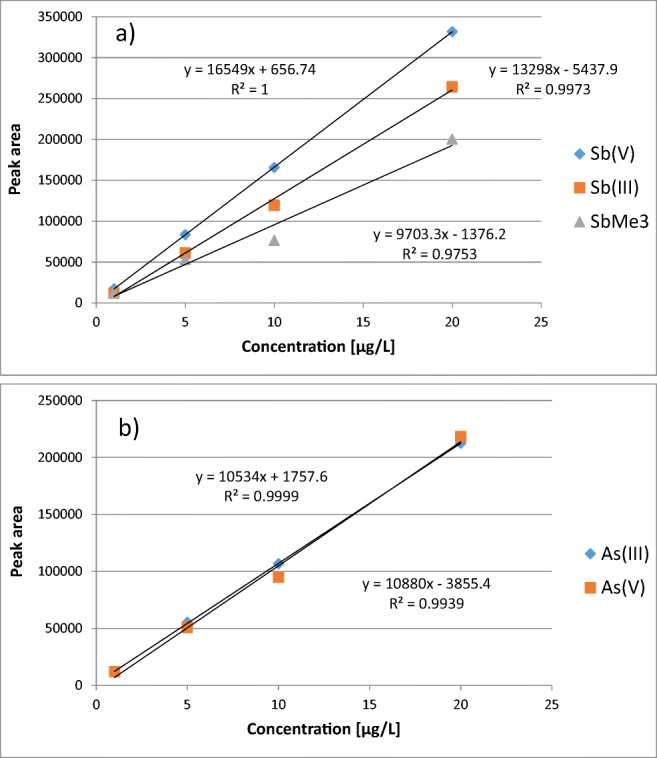
Calibration curves **a** antimony and **b** arsenic species

The standards of arsenic and antimony species were together in the same solution during calibration preparation. Calibration curves were obtained with measurement of 1 μg/L, 5 μg/L, 10 μg/L, 20 μg/L standard solutions for As(III), As(V), Sb(III), Sb(V), and SbMe_3_, respectively. A linear model of the dependence of concentration of the total number of analyte counts was selected. The coefficient of determination of calibration curves *R*^2^ was between 0.9753 and 0.9999. Figure 2 presents superimposed chromatograms obtained after analyzing As and Sb standard solutions and Fig. 3 shows calibration curves arsenic and antimony species.

### Sequential chemical extraction

The BCR (the Institute for Reference Materials and Measurements) sequential chemical extraction (Fadiran et al. 2014) helped to determine the arsenic and antimony forms in the bottom sediments and the way in which they were bound. Sequential chemical extraction included stages: F1 mobile exchangeable fraction extracted with acetic acid, associated with adsorbed cations and anions on sediment, and carbonates, and very reactive oxy-hydroxides; F2 mobile reducible fraction extracted with hydroxyl ammonium chloride, associated with iron/manganese oxides; F3 immobile oxidizable fraction extracted with ammonium acetate and perhydrol, associated with organic substance and sulfides; F4 immobile residual fraction extracted with *aqua regia* associated with non-silicate bound metals.

### Assessment of the Kozłowa Góra Reservoir sediment contamination

The evaluation of the bottom sediment pollution was performed using four methods: Sb/As factor, which is one of the indicators of pollution origin sources (Fu et al. 2011; Bi et al. 2011; Sharifi et al. 2016), Geoaccumulation Index (*I*_geo_) proposed by Müller (1969), Pollution Load Index (Tomlinson et al. 1980), and LAWA classification (LAWA 1998).

Geoaccumulation Index (I_geo_) is defined as

1 $$ {I}_{geo}= lo{g}_2\left({C}_{EL}/1.5\ {C}_{background}\right) $$

where *C*_EL_ is the total element concentration in the bottom sediment samples, *C*_background_ is the geochemical background of element concentration, factor 1.5 is the correction factor compensating for natural (lithological) variations in geochemical data. *I*_geo_ describes the pollution of the bottom sediments by an element with respect to seven classes from 0 to 6. The class 0 (*I*_geo_ ≤ 0) belongs to non-polluted sediments. First class (0 < *I*_geo_ ≤ 1) describes uncontaminated, to the moderately contaminated sediments. Second class (1 < *I*_geo_ ≤ 2) is moderately contaminated sediments. The third class (2 < *I*_geo_ < 3) concerns sediments moderately or heavily polluted. To the fourth class (3 < *I*_geo_ < 4), there are heavily contaminated sediments. Then, the fifth class (4 < *I*_geo_ < 5) belongs heavily to very heavily polluted sediments. And the last one is sixth class (*I*_geo_ > 5)—very contaminated sediments.

Pollution Load Index (PLI) is often used to determine the level of the bottom sediment contamination compared to background concentration levels (Lis and Pasieczna 1998). To calculate the PLI, the following equations were used:

2 $$ C{F}_{EL}={C}_{EL}/{C}_{background} $$

3 $$ PLI={\left(C{F}_{EL1}\times C{F}_{EL2}\times C{F}_{EL3}\times \dots \dots \times C{F}_{EL n}\right)}^{1/n} $$

where CF_EL_ is the pollution factor defined as the ratio of the element concentration in the bottom sediment to the concentration of this element in the background.

The assessment of the Kozłowa Góra Reservoir bottom sediment quality was also carried out in accordance with the LAWA classification (LAWA 1998). In this classification, the bottom sediments are assessed in terms of metal content in four classes: first class un-polluted sediments, second class moderately contaminated sediments, third class heavily contaminated deposits, and fourth class very heavily polluted sediments.

## Results and discussion

### Speciation analysis method

In this work, the Dionex IonPac AS7 column was used for the first time, for the simultaneous determination of five ionic arsenic and antimony forms [As(III), As(V), Sb(III), Sb(V), SbMe_3_].

The methodology development involved mainly optimization of separation conditions, including the mobile phase, flow time, volume of injected sample, and temperature. The choice of the mobile phase was dictated by previous experiments, in which inorganic antimony ionic forms [Sb(III) and Sb(V)] were determined in waters and bottom sediments of Polish rivers (Jabłońska-Czapla and Szopa 2016). One of the important parameters was the optimization of the eluent pH, from pH = 4.5 (Jabłońska-Czapla and Szopa 2016) to pH = 4.0. The used mobile phase (phthalic acid and Na_2_EDTA) is ideal for separating both the antimony and arsenic ionic forms. The analysis time was one of the key parameters, because we wanted to simultaneously determine five ionic forms in the shortest possible time of analysis. Optimal separation conditions were obtained within 6 min.

The calibration curves (Fig. 3) were obtained through measuring the standard solutions (concentrations of 1 μg/L, 5 μg/L, 10 μg/L, and 20 μg/L) for five speciation arsenic and antimony forms. The linear model of the concentration dependence on total analyte counts was selected. The obtained curves had good compatibility. Using the numerous determinations of the calibration curves, they also helped to calculate the limit of detection (LOD) for the arsenic and antimony speciation forms. The LOD calculation was based on the following dependence:

4 $$ LOD=\frac{3\ast s}{b} $$

where *s* is the standard deviation value, *b* is the slope of a straight calibration line.

The standard deviation value could be determined as the standard deviation of the offset of the obtained calibration curve. The limits of detection are given in Table 3. The optimized method is selective and demonstrates low detection limits. The repeatability, intermediate precision, and accuracy allow for its use in the trace analysis of environmental samples.

### Validation of methodology

In order to check the methodology of total metals and metalloid determination in the water and bottom sediment samples, the certified reference material analysis was used: NCS DC 73309, NCS DC 73310, NCS DC 73312 (China National Analysis Center for Iron and Steel) as well as Certified Reference Material NIST1643-e (National Institute of Standard and Technology). Table 2 presents the validation parameters of the total metal and metalloid analysis, recovery in CRMs using the ICP-MS technique.

**Table 2:** Validation parameters of the total metal and metalloid analysis, recovery of CRMs using the ICP-MS technique

Analyte	LOD [μg/L]	LOQ [μg/L]	U [%]	NCS DC 73309			NCS DC 73310			NCS DC 73312			NIST 1643e		
				CRM [mg/kg]	Measured value [mg/kg]	*R* [%]	CRM [mg/kg]	Measured value [mg/kg]	*R* [%]	CRM [mg/kg]	Measured value [mg/kg]	*R* [%]	CRM [μg/L]	Measured value [mg/kg]	*R* [%]
Mn	0.03	0.1	10	2490 ± 84	2451 ± 249	98	1400 ± 47	1436 ± 144	103	240 ± 20	246 ± 25	102	38.97 ± 0.45	36.48 ± 3.6	94
Co	0.07	0.19	10	8.5 ± 0.8	8.19 ± 0.82	96	8.8 ± 0.7	8.31 ± 0.83	95	2.6 ± 0.7	2.04 ± 0.2	79	27.06 ± 0.32	26.15 ± 2.6	97
Ni	0.11	0.33	21	14.3 ± 1	14.78 ± 3	103	12.8 ± 1.3	12.49 ± 2.6	98	5.5 ± 1.4	4.59 ± 0.96	83	62.41 ± 0.69	59.051 ± 12	95
Cu	0.11	0.33	10	79 ± 3	79.4 ± 7.9	100	1230 ± 33	1231 ± 123	100	4.9 ± 0.5	4.29 ± 0.429	87	22.76 ± 0.31	20.039 ± 2	88
Zn	0.18	0.54	31	373 ± 14	310 ± 96	83	498 ± 18	430 ± 133	86	44 ± 5	13.5 ± 4.2	81	78.5 ± 2.2	48.54 ± 15	62
As	0.09	0.27	29	188 ± 13	190 ± 55	101	115 ± 6	115 ± 33	100	6.2 ± 0.6	5.4 ± 1.6	88	60.45 ± 0.72	52.85 ± 15	87
Rb	0.01	0.03	14	408 ± 11	540 ± 76	132	270 ± 10	350 ± 49	130	470 ± 23	379 ± 81	81	14.14 ± 0.18	15.58 ± 2.2	110
Sr	0.01	0.02	11	29 ± 4	23.4 ± 2.6	81	24 ± 3.0	20.9 ± 2.3	87	ND	ND	ND	323.1 ± 3.6	348.5 ± 38	108
Ag	0.01	0.03	10	3.2 ± 0.4	3.4 ± 0.34	108	1.15 ± 0.11	1.53 ± 0.15	133	0.066 ± 0.01	0.087 ± 0.01	132	1.062±0.07	1.027 ± 0.1	97
Cd	0.01	0.04	10	2.3 ± 0.2	1.8 ± 0.18	80	4.0 ± 0.3	4.13 ± 0.41	103	0.065 ± 0.01	0.063 ± 0.01	97	6.568 ± 0.07	5.94 ± 0.59	90
Ba	0.1	0.3	15	260 ± 17	175 ± 26	67	206 ± 15	160 ± 24	78	185 ± 24	87.0 ± 13	87	544.2 ± 5.8	504 ± 71	92
Tl	0.01	0.03	28	2.9 ± 0.4	3.41 ± 0.9	118	1.76 ± 0.27	1.63 ± 0.46	93	1.9 ± 0.4	1.88 ± 0.53	99	7.445 ± 0.1	7.91 ± 2.2	106
Pb	0.09	0.28	12	636 ± 22	659 ± 79	104	285 ± 11	296 ± 35.5	104	32 ± 5	31.8 ± 3.8	99	19.63 ± 0.21	20.23 ± 2.4	121
Cr	0.11	0.34	23	40 ± 3	35.5 ± 8.1	89	35 ± 3	25.6 ± 5.89	73	12 ± 3	8.11 ± 1.9	68	20.40 ± 0.24	21.33 ± 4.9	104
Sb	0.01	0.03	28	14.9 ± 1.2	15.0 ± 4.2	101	24 ± 3	25.4 ± 7.1	106	0.46 ± 0.12	0.66 ± 0.18	103	58.30 ± 0.61	56.83 ± 15	97

*LOD* limit of detection, *LOQ* limit of quantification, *CRM* the element content from the certificate of reference material, *U* uncertainty, *R* recovery, *ND* no data

Measured value-result of the elemental analysis of reference material after complete digestion using the ICP-MS technique

The extraction efficiency of arsenic and antimony from the bottom sediments was checked by analyzing bottom sediment CRM (NCS DC 73310). The extraction of certified reference material with phosphate buffer showed that yield for arsenic was 37%, while extraction efficiency of antimony from Na_2_EDTA solution was 32%. These extraction conditions do not affect the ionic form of the element, which has been checked previously (Jabłońska-Czapla and Szopa 2016).

Basic validation parameters for water were determined for the simultaneous determination of five arsenic and antimony ionic forms by the standard addition method, and the obtained results are presented in Table 3.

**Table 3:** Validation parameters of simultaneous determination of As(III), As(V), Sb(III), Sb(V), and SbMe_3_ species using HPLC-ICP-MS

Analyte	Retention time [min]	LOD [μg/L]	The recovery by standard addition method [%]	RSD [%]	Uncertainty [%]
As(III)	1.84	0.09	94	3.2	15
As(V)	3.77	0.12	98	3.3	17
Sb(III)	3.47	0.009	99	3.1	12
Sb(V)	2.25	0.014	101	2.9	13
SbMe_3_	4.28	0.027	105	3.5	15

*LOD* limit of detection, *RSD* relative standard deviation

### Basic physicochemical parameters

**Fig. 4:**
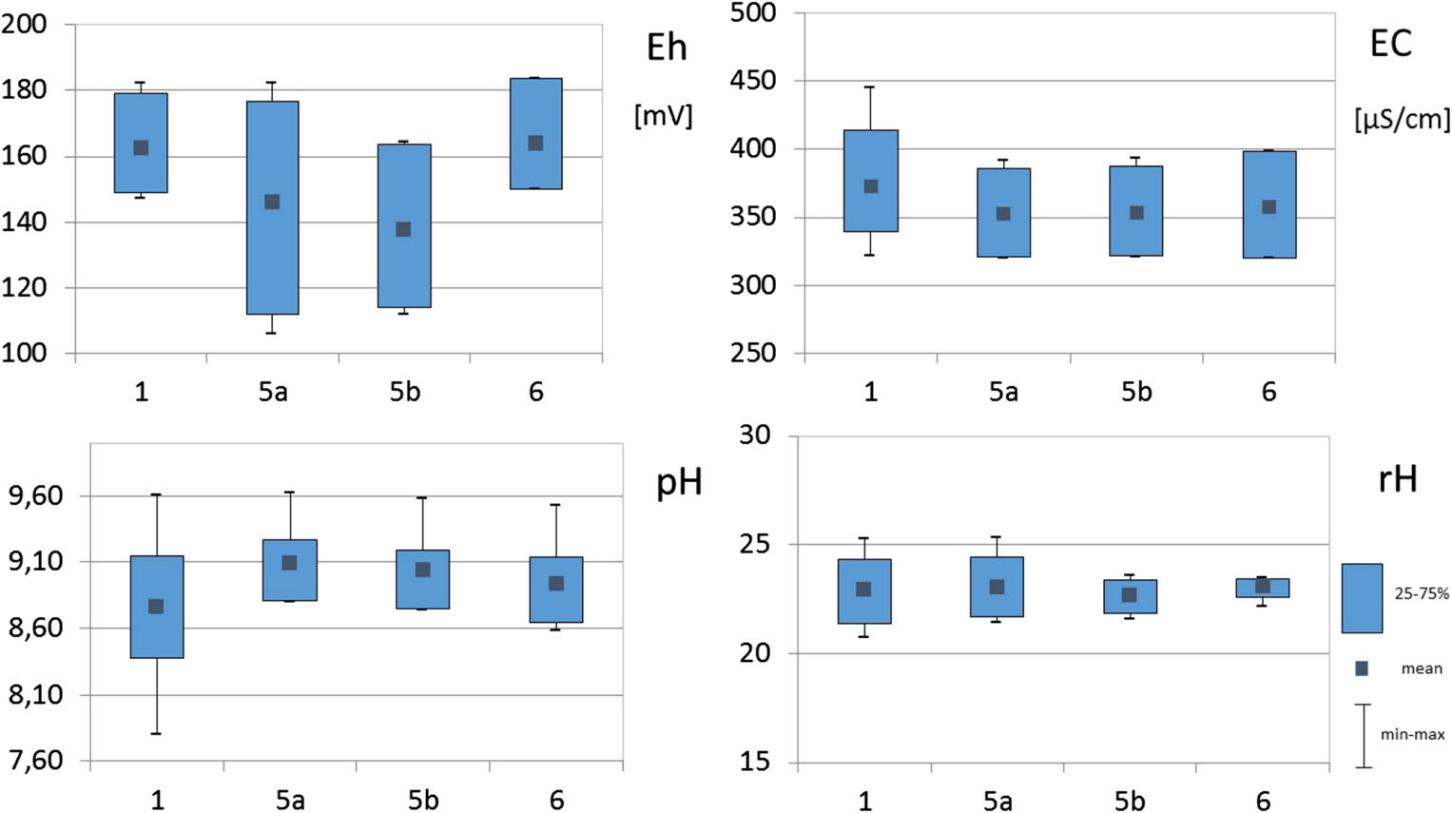
Variability of physicochemical parameters of Kozłowa Góra Reservoir water; Eh–redox potential, EC–conductivity, rH–Clark coefficient

Water samples were collected at three points: at the mouth of the Brynica River to the Kozłowa Góra Reservoir, from the reservoir in the dam area (surface and bottom water), as well as from the Brynica River behind the reservoir (see Fig. 1). Figure 4 graphically presents the variability of physicochemical parameters of water. The basic physicochemical parameters of water have shown a high variability in the redox potential, especially in the case of water collected in the dam zone. Surface water at this sampling point has Eh value from minimum 105.8 mV to a maximum 182.2 mV. However, the values of the bottom water redox potential were at least 111.8 mV and a maximum of 164.4 mV, respectively. Minor variations in this parameter occurred in the water flowing in and out of the tank. The highest variability of the pH value was shown in the water samples flowing into the reservoir, which is probably related to the variable composition of the Brynica River flowing into the Kozłowa Góra Reservoir. Despite significant differences in the redox potential as well as the hydrogen ion concentration, the Clark coefficient calculation showed that water exists in oxidizing conditions, with an average value of rH parameter equal 23. The water conductivity (EC) in the reservoir showed the highest values at the first sampling point (maximum 445 μS/cm), at the mouth of the Brynica River into the reservoir. This indicates a large charge of ions introduced through this watercourse into the Kozłowa Góra Reservoir. In the Clark scale, the reduction values occur at rH < 15, while oxidizing conditions at rH > 25 (Clark 1923; Drobnik and Latour 2003). The bottom sediments pH value showed variation in the range of 6.73–7.57 (Table 4). Calculated rH coefficient was < 15 that indicated reduction condition in the Kozłowa Góra Reservoir bottom sediments.

**Table 4:** Basic physicochemical parameters of the Kozłowa Góra Reservoir bottom sediments

Date of sampling	Sampling point	pH	Eh [mV]	rH
May	1	7.50	− 191	9
	2	7.41	− 304	5
	3	7.20	− 309	9
	4	6.96	− 278	5
	5	7.12	− 312	4
June	1	6.93	− 284	4
	2	7.08	− 287	5
	3	7.26	− 308	4
	4	7.49	− 277	6
	5	7.52	− 261	6
July	1	7.46	− 275	6
	2	7.51	− 272	6
	3	7.20	− 303	4
	4	7.10	− 294	4
	5	7.54	− 305	5
August	1	7.37	− 280	5
	2	7.57	− 302	5
	3	7.20	− 306	4
	4	7.03	− 309	4
	5	7.04	− 295	4
September	1	7.31	− 214	7
	2	7.38	− 297	5
	3	7.12	− 244	6
	4	7.07	− 223	7
	5	6.73	− 257	5

### Total metal(loid) concentration

#### Water

According to the Polish regulation about acceptable metal(loid) content for surface water (Regulation of Minister of Environment on 21 July 2016 on the Classification Status of Surface Waters and Environmental Quality Standards for Priority Substances), the Kozłowa Góra Reservoir water should be included to the I and II class of surface water purity. Additionally, reservoir water (in terms of metal and metalloid content) meets the requirements for drinking water (Regulation of the Minister of Health on 7 December 2017 on the Quality of water intended for human consumption). Table 5 presents the minimum, maximum, and mean metal(loid) contents in the Kozłowa Góra Reservoir water and bottom sediment samples.

**Table 5:** Total minimum (Min), maximum (Max), and median metal(loid) concentration in the water and bottom sediment of the Kozłowa Góra Reservoir

Analyte	Water *N* = 20			Bottom sediment *N* = 25		
	Min [μg/L]	Max [μg/L]	Median [μg/L]	Min [mg/kg]	Max [mg/kg]	Median [mg/kg]
Mn	45.2	262.5	139.5	433.5	1258	786.7
Co	0.18	1.00	0.24	2.56	27.0	15.9
Ni	1.77	4.10	2.22	2.10	41.1	26.5
Cu	0.79	7.26	1.27	14.75	74.7	44.8
Zn	3.68	71.83	9.92	994.7	3764	2632
As	1.66	2.37	1.96	18.97	60.7	33.4
Rb	3.80	5.03	4.76	26.9	38.7	32.2
Sr	97.8	128.1	110.7	43.8	92.5	75.2
Cd	0.06	1.13	0.16	4.99	27.3	22.5
Ba	92.3	156.9	116.2	399.2	1064	555.34
Tl	<LOD	0.13	<LOD	0.49	2.21	1.37
Pb	1.52	16.9	3.01	149.4	679.1	391.6
Sb	0.67	1.58	1.01	0.32	3.30	1.86
Cr	31.4	79.1	55.7	79.0	31.4	55.7

*N* number of samples, *LOD* limit of detection

**Fig. 5:**
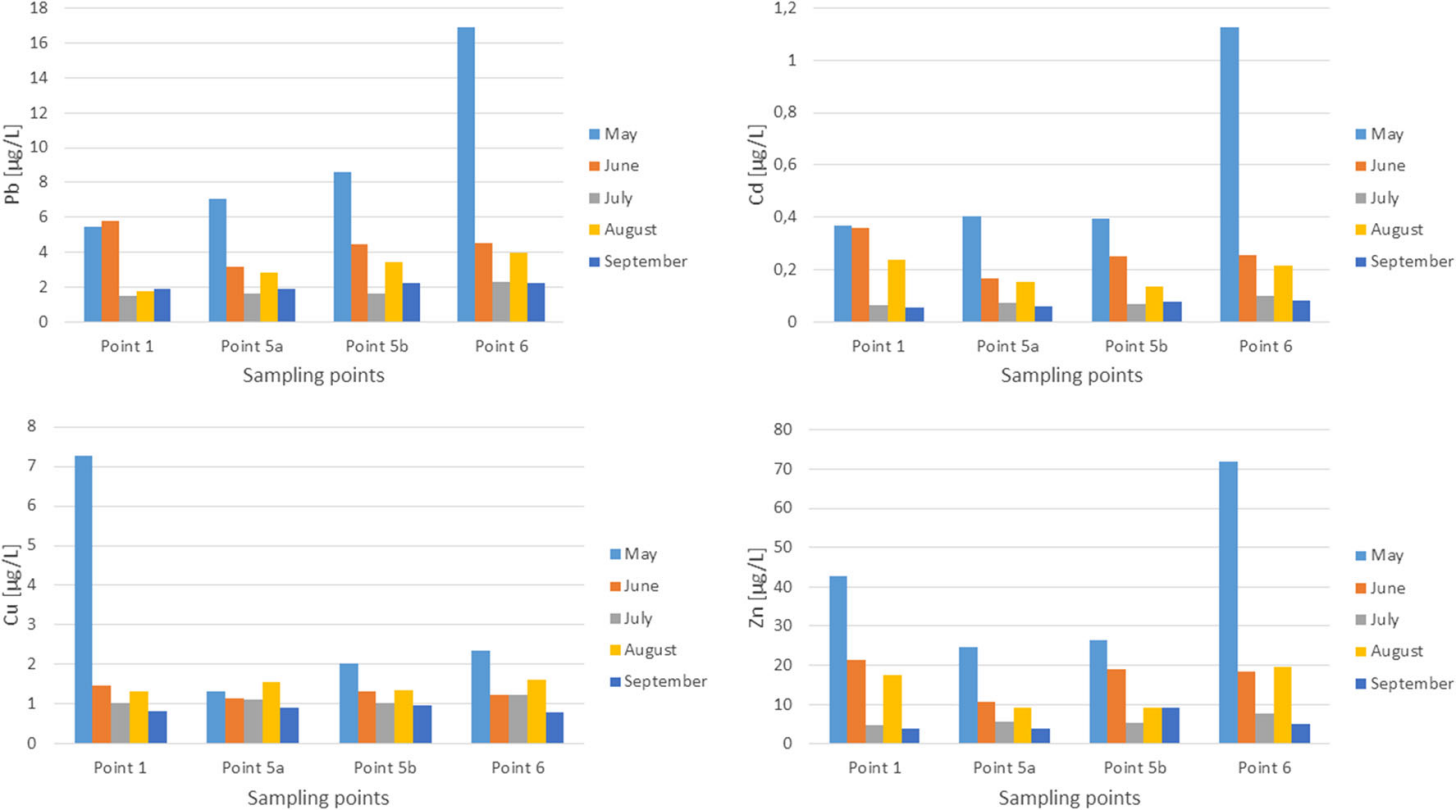
Total lead, cadmium, zinc, and copper concentrations in the Kozłowa Góra Reservoir water; sampling points 1–the inflow area of the Brynica River to the reservoir, 5a–the dam zone, surface water, 5b–the dam zone, bottom water, 6–Brynica River–outflow, below the dam

Total arsenic concentration in the reservoir water did not show significant temporal or spatial variation. The arsenic concentration increased slightly in the season and the highest values occurred in September. Only in May, the concentration of arsenic increased in the transect 1-5a-5b-6, so that the highest concentration occurred at the mouth of the water from the reservoir to the Brynica River. In the case of antimony, similarly to arsenic, it was characterized by low variability of concentrations. The highest antimony concentration occurred in August in surface water at the first sampling point. The thallium concentration was extremely low and amounted to 0.134 μg/L. The highest concentration of this element occurred in the water flowing into the reservoir and was related to the pollution of Brynica River. The concentration of lead, zinc, and cadmium in the water increased in the transect along the long axis of the reservoir (Fig. 5). The highest concentrations of these elements occurred at the last sampling point, at the mouth of the water from the reservoir to the Brynica River. Increased concentration of these elements may also be associated with the precipitation of dust, industrial dust, and traffic. The highest concentration of lead, cadmium, and zinc in reservoir water occurred in May. The concentration of copper was in the range of 0.79–7.26 μg/L, with the highest concentration of this element occurring in May at the first sampling point. A high zinc concentration occurred at the first sampling point, which was related to the introduction of contaminants by the Brynica River flowing into the reservoir. Then, the zinc concentration decreased in the tank’s transect to increase again at the 6th water sampling point, at the mouth of the Brynica River from the reservoir. In the case of copper, the trend was similar, except for one subscription in May, when the copper, manganese, cobalt, nickel concentrations suddenly rose in a water sample which was most likely caused by a discharge of sewage into the Brynica River flowing into the reservoir.

#### Bottom sediments

**Fig. 6:**
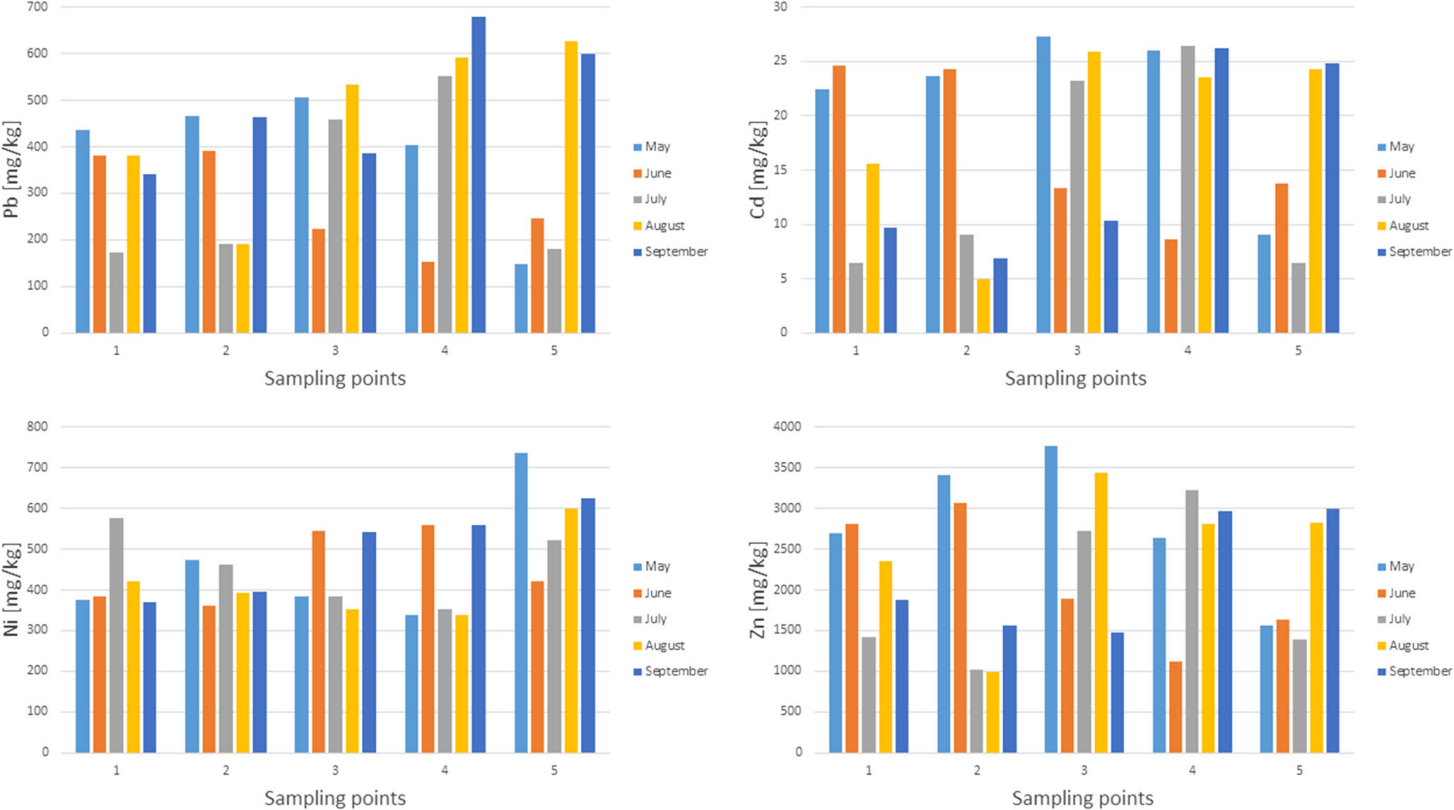
Total lead, cadmium, zinc, and nickel concentrations in the Kozłowa Góra Reservoir bottom sediments; sampling points 1–the inflow area of the Brynica River to the reservoir; 2, 3, 4–points lying in the transect; 5–the dam zone

As demonstrated by previous research (Dąbrowska 2011), the bottom sediments of the Kozłowa Góra Reservoir consist mainly of granular fraction: < 0.25 mm (30–42%) and 0.25–0.5 mm (29–37%), represented mainly by medium sand and fine sand, dust and loam (Particle Size Distribution and Textural Classes of Soils and Mineral Materials 2009). Moreover, bottom sediments are characterized by significant contamination with heavy metals (Rosińska and Dąbrowska 2011). Figure 6 shows the total lead, zinc, cadmium, and nickel content in the bottom sediment samples.

The reservoir sediments are heavily contaminated by lead in which average value was 391 mg/kg, and in September at the fourth sampling point (in the middle of the tank); its concentration was even 679 mg/kg.

Studies have shown that bottom sediments of Kozłowa Góra Reservoir are strongly polluted by zinc and cadmium, and the highest concentration of these elements was recorded in May, in the middle of the reservoir at the third collection point, and was 3800 mg/kg and 22.49 mg/kg, respectively.

Based on the geochemical criteria assessment (Siebielec et al. 2015), the bottom sediments of the Kozłowa Góra Reservoir should be classified as heavily polluted with lead, cadmium, zinc, and moderately contaminated with arsenic and nickel. The average nickel concentration in the bottom sediment was 21 mg/kg, whereby the maximum concentration of this element was determined in August in the middle of the tank (41 mg/kg). This element gets to the reservoir bottom sediments mainly from anthropogenic sources. In the bottom sediments, there is a small spatial variability of metal and metalloid concentrations. In the case of lead, increase in this element concentration along a transect was observed, with the highest levels observed in the middle of the tank (third and fourth sampling point) (Fig. 6). The zinc, lead, arsenic, antimony, and copper concentration in the bottom sediments also increased in the transect, so that the highest values were shown in the middle of the tank. The migration of metals along the reservoir transect is closely related to its morphometry.

### Speciation of antimony and arsenic in the water and bottom sediment of Kozłowa Góra Reservoir

#### Antimony speciation

**Fig. 7:**
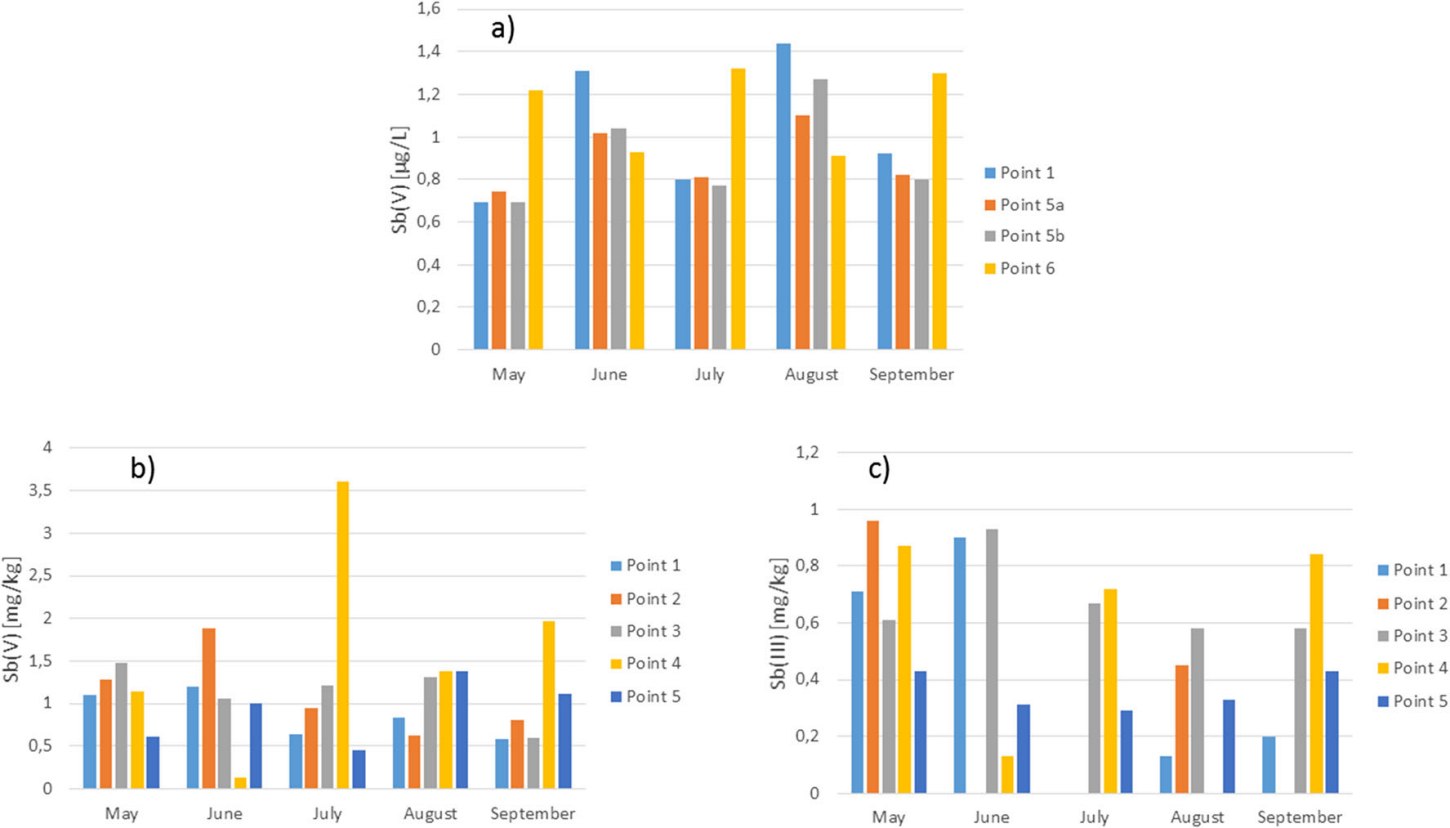
Antimony speciation using HPLC-ICP-MS techniques **a** in the water and **b**, **c** the bottom sediment of Kozłowa Góra Reservoir

In water of this anthropogenic reservoir, oxidation conditions have always occurred during the whole research period, as indicated by the high Clark factor. These conditions have a strong impact on the occurrence of only the oxidized antimony form Sb(V) in the reservoir waters (Fig. 7).

Research has shown that the Sb(III) concentration was below the limit of determination (< 0.009 μg/L). While the average Sb(V) concentration was 0.93 μg/L, the highest ion concentration occurred in August at the mouth of the estuary Brynica River to the reservoir [Sb(V) = 1.44 μg/L].

A strong correlation between the Sb(V) concentrations in surface and bottom water was found [*R*^2^_Sb(V)surface/Sb(V)bottom_ = 0.972], which was caused by a similar concentration of this ion in surface and benthic water. This is due to the fact that it is a flow tank, in which the masses of water mix, and the concentrations of trace elements, like antimony are averaged. The presence of a methyl antimony derivative was not found in the Kozłowa Góra Reservoir water. In most cases, the main mediator of the antimony biomethylation process is microorganisms (Jenkins et al. 1998). Methyl antimony derivatives can be brought into the environment by human activity or as a result of methylation processes (Sun et al. 1993). The antimony biomethylation mechanism is similar to arsenic, but it is not fully understood and still under investigation. Duan et al. (2010) found that Bohai Bay’s water contained mainly Sb(V) form, and its share in relation to the total antimony was 89%. However, our previous investigations have shown that there may be a reduced antimony form in the dam reservoir water and that the Sb(III) concentration increases in the bottom water (Jabłońska-Czapla et al. 2015). In the Kozłowa Góra Reservoir conditions, there was no reduced antimony form, also in the bottom water. The obtained results confirmed the literature data that the high Sb(III) concentration is characteristic of surface water with low oxygen content, whereas Sb(V) concentration is more strongly dependent on pH and Eh changes (He et al. 2019).

In the Kozłowa Góra Reservoir bottom sediments, there were both inorganic antimony species, in which concentration significantly correlated (*R*^2^_Sb(V)/Sb(III)_ = 0.849), while there was no organic antimony (SbMe_3_) form. Literature reports show that antimony methyl derivatives were found in river bottom sediment samples (Krupp et al. 1996) or in bottom sediment samples studied by Duester et al. (2007). In the Kozłowa Góra Reservoir, the concentration of antimony and its species in the bottom sediments was the highest at the central sampling points and decreased just at the dam zone. The highest concentration of oxidized antimony form occurred in July to the middle of the tank and was Sb(V) = 3.61 mg/kg. The reduced form of this element was much lower in the bottom sediments, on average 0.43 mg/kg. In the bottom sediments, a strong correlation was found between Sb(V) and Sb(III) concentration and at the first sampling point just at the inflow of Brynica River waters to the reservoir it was *R*_1_^2^_Sb(V)/Sb(III)_ = 0.849. The obtained results indicate the influence of physicochemical conditions on changes in the antimony ionic form concentration. The concentration of Sb(V) correlated strongly with pH (*R*_2_^2^_pH/Sb(V)_ = 0.828) and Eh (*R*_3_^2^_Eh/Sb(V)_ = 0.814).

#### Arsenic speciation

**Fig. 8:**
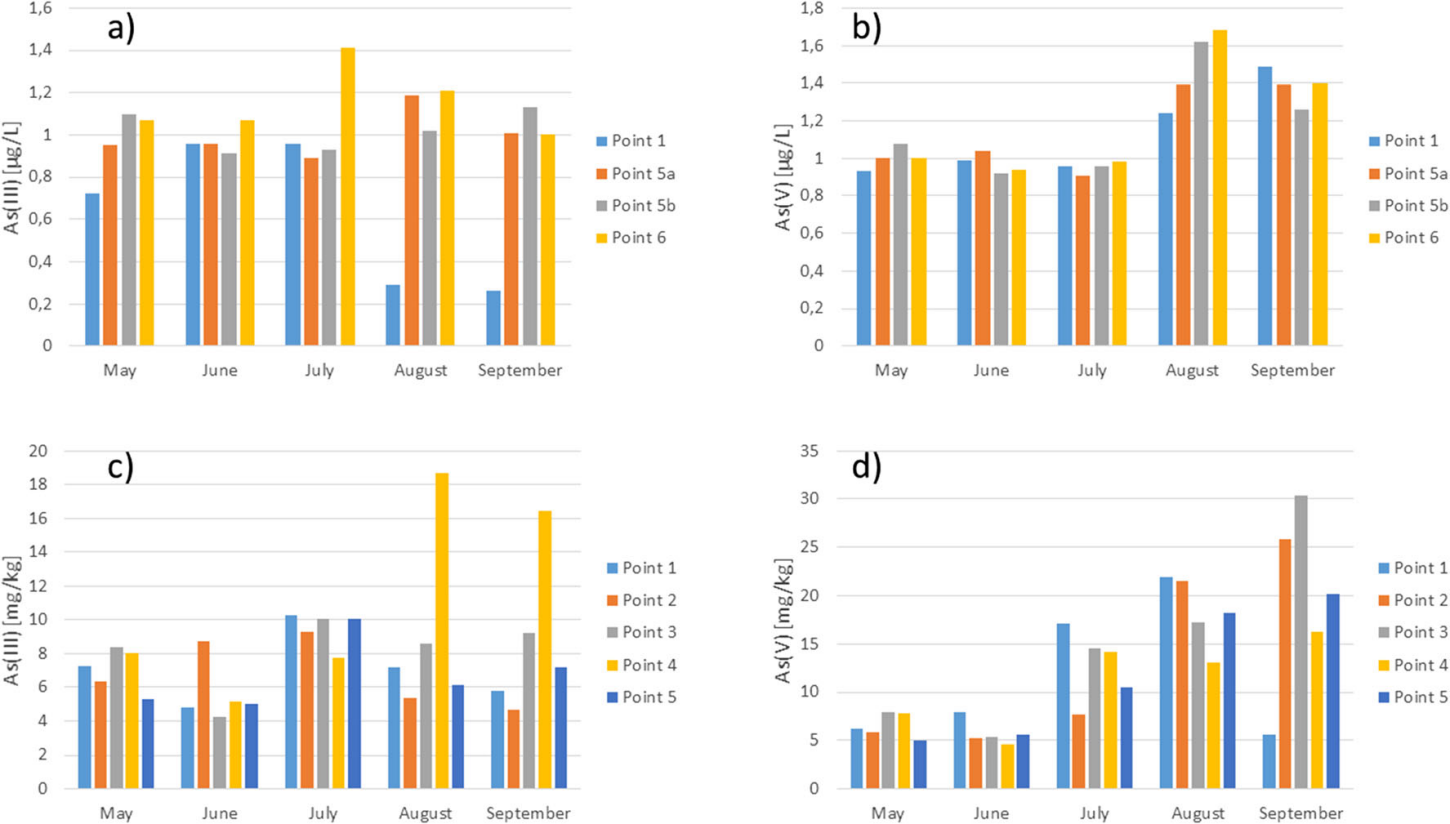
Arsenic speciation using HPLC-ICP-MS techniques **a**, **b** in the water and **c**, **d** the bottom sediments of Kozłowa Góra Reservoir

Figure 8 presents the results of arsenic speciation analysis in the water and bottom sediments. In the Kozłowa Góra Reservoir waters, there were both inorganic arsenic forms and in comparable concentrations. Similar results were obtained in the Rybnik Reservoir research (Jabłońska-Czapla et al. 2015). In the case of Rybnik Reservoir, the bottom waters were richer in the reduced arsenic form. However, as shown in the research, the Kozłowa Góra Reservoir bottom water did not contain large amounts of the reduced arsenic form. Both in the upper and lower layers of water taken at fifth sampling point (5a and 5b) concentrations of As(III) and As(V) were comparable. Water of the Kozłowa Góra Reservoir contained an average 0.98 μg/L As(III) and 1.02 μg/L As(V). Similarly to antimony, a strong correlation was found between the As(V) concentrations in the water sampling at point 5a and 5b (*R*^2^_As(V)surface/As(V)bottom_ = 0.724), which was caused by a similar concentration of this ion in surface and bottom water. In addition, there was a strong correlation between As(V) and As(III) concentrations in surface water at the first and fifth sampling points: *R*_1_^2^_As(V)/As(III)_ = 0.724 and *R*_5a_^2^_As(V)/As(III)_ = 0.686, respectively. Due to permanent oxygen conditions in the Kozłowa Góra Reservoir, contrary to the conditions prevailing in the Pławniowice reservoir (Jabłońska-Czapla et al. 2014), no drastic changes in the concentration of both ionic arsenic forms were observed.

In the bottom sediments, the average arsenic content was 33 mg/kg, which according to the geochemical classification (Siebielec et al. 2015), means pollution of the Kozłowa Góra Reservoir with arsenic and classifies its sediments to II–III class of moderately polluted and contaminated bottom sediments. The bottom sediments contained more As(V). The concentration of both arsenic ionic forms in bottom sediments was comparable in May and June. The maximum concentration of toxic As(III) (18.7 mg/kg) occurred in August in the fourth sampling point in the middle of the tank, but otherwise, the concentration of this ionic form was lower and amounted to about 10 mg/kg. The highest concentration of this ionic form occurred in the middle of the tank. The As(III) concentration grew along the transect, and just at the dam dropped. Kozak et al. (2007) examining the Winiary and Jelonek Lakes, which were subject to urban anthropopression, and found that in the bottom sediments of these reservoirs, the easily-leached sediment fraction was richer in As(III). Our research has shown that the easy-leached bottom sediment fraction of the Kozłowa Góra Reservoir contained in most cases more As(V), but there were also samples in May, when the concentration of the reduced form of arsenic was higher. The bottom sediments of the Pławniowice Reservoir were also richer in As(V) (Jabłońska-Czapla et al. 2014). In the water reservoir, the predominance of the oxidized arsenic form As(V) is a desirable and favorable phenomenon for the demobilization of arsenic in the bottom sediments. The concentration of oxidized arsenic form in the sediments was characterized by strong variability in time, increased in the season, with the highest value in September at the third sampling point in the middle of the reservoir. In the bottom sediments of the reservoir, the reduction conditions prevailed, but water was characterized by good oxygen conditions. The anoxic environment helps to reduce arsenates to arsenites. As passed into the bottom sediments and, depending on the redox conditions, could transform into soluble arsenites, insoluble sulfides, or free As (Orero Iserte et al. 2004). Good aerobic conditions of the Kozłowa Góra Reservoir limit the above-mentioned processes, which results in the reduction of the ecological hazard associated with the sudden release of ionic arsenic forms. The norm on the quality of water intended for human consumption (Regulation of the Minister of Health on 7 December 2017 on the Quality of water intended for human consumption) states that the maximum concentration of As may be up to 10 μg/L and the Kozłowa Góra Reservoir water meet these requirements. On the other hand, the reservoir sediments contained average about 33 mg/kg of arsenic (maximum 60.69 kg), including high content of toxic As(III), which when the physicochemical conditions change (i.e., pH or redox potential), it is possible to release the deposited toxic As(III) to the water.

### Sequential chemical extraction

**Fig. 9:**
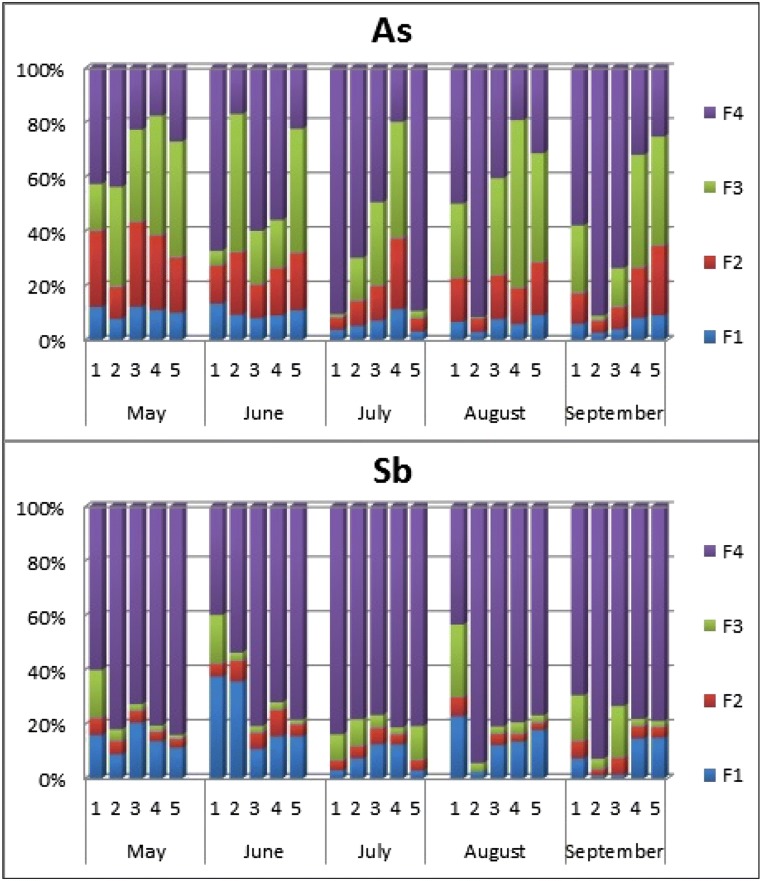
Sequential chemical extraction of the bottom sediment, F1–mobile exchangeable fraction; F2–mobile reducible fraction (associated with iron/manganese oxides); F3–immobile oxidisable fraction (associated with organic substance and sulfides); F4–immobile residual fraction (associated with non-silicate bound metals), 1–5–the bottom sediment sampling points

The BCR (the Institute for Reference Materials and Measurements) (Fadiran et al. 2014) chemical extraction of the bottom sediments was carried out. This allowed the separation of metals associated with the three main fractions and an additional residual fraction. This work focuses on the sequential chemical extraction of arsenic and antimony because in the available literature on research of the Kozłowa Góra Reservoir, the results of tests for Zn, Cu, Ni, Cd, Pb, and Cr were found (Dąbrowska 2011; Rosińska and Dąbrowska 2011). Figure 9 shows the results of sequential chemical extraction of the bottom sediment for arsenic and antimony. Arsenic in the Kozłowa Góra Reservoir bottom sediments is mainly associated with sulfides and organic matter (F3) and deposited in the residual fraction (F4).

The smallest share of arsenic in bottom sediments occurred in the ion exchange fraction. Only a few times during the research period the share of arsenic in mobile fractions was around 40% (May, the first and third sampling point; July, the fourth point; September, the fifth sampling point of bottom sediments).

Antimony in the reservoir bottom sediments was largely deposited in the residual fraction (F4). Only at the first sampling point, the residual fraction share was slightly smaller and amounted to approx. 40%. In other cases, regardless of the place or time of sampling, the antimony share in the residual fraction was the highest and averaged 80%.

While the share of antimony in the reducible fraction was the smallest, it means that antimony was not bound in the bottom sediment with iron and manganese oxides. From the mobile fractions, to the greatest extent (about 40%), antimony in the bottom sediments was bound with the ion exchange fraction in July at the first and a second sampling point, except for the second and third sampling point in September.

### Assessment of the bottom sediment contamination of the Kozłowa Góra Reservoir

#### Sb/As coefficient

Sb/As factor is one of the indicators of pollution origin sources (Bi et al. 2011; Fu et al. 2011; Sharifi et al. 2016). The Sb/As coefficient was determined as part of the Kozłowa Góra Reservoir bottom sediment study. The average value for all tested bottom sediment samples was Sb/As = 0.06. The results indicate the occurrence of the communication emission, hard coal combustion, smelting Zn-Pb ores impact on the bottom sediment contamination.

#### LAWA classification

The assessment of the Kozłowa Góra Reservoir bottom sediment quality was carried out in accordance with LAWA classification (LAWA 1998). The obtained results indicate that the reservoir bottom sediments are heavily contaminated. In terms of copper content (average 44.8 mg/kg), the bottom sediments can be classified as moderately contaminated. However, in the case of the zinc (average of 2632 mg/kg) and lead content (average of 391 mg/kg), the reservoir sediments are heavily contaminated with the above-mentioned metals and are classified in the III class of purity. In addition, in the Kozłowa Góra Reservoir, very high concentrations of cadmium (on average 22.5 mg/kg) occur, and the bottom sediments are classified as IV class of purity—very heavily contaminated sediments.

#### Geoaccumulation index (*I*_geo_)

Table 6 shows the results of the *I*_geo_ calculations. The bottom sediments of the Kozłowa Góra Reservoir are extremely contaminated by Zn and Pb (6th class *I*_geo_), followed by Cd, Cr, Ba (5th class *I*_geo_), which according to Müller classification (1969) indicates that deposits are heavily contaminated with these elements. However, due to arsenic pollution, the bottom sediments are moderately or heavily polluted (3rd class *I*_geo_).

**Table 6:** Geocumulation Index (*I*_geo_) of the Kozłowa Góra Reservoir bottom sediments

Date	Sampling points	Mn	Co	Cu	Zn	As	Sr	Cd	Ba	Pb	Sb	Cr
May	1	2.83	3.68	3.32	5.95	1.92	1.87	4.91	4.55	5.38	0.94	3.94
	2	2.92	4.04	3.96	6.29	2.30	2.14	4.98	5.47	5.47	1.86	4.44
	3	3.05	4.17	3.99	6.44	2.31	2.14	5.18	5.14	5.59	2.14	4.39
	4	2.49	3.44	3.56	5.92	1.93	1.95	5.11	4.47	5.26	1.34	4.33
	5	2.53	2.54	1.86	5.16	1.34	1.48	3.59	4.46	3.83	0.00	3.39
June	1	2.69	3.65	3.46	6.01	1.91	1.97	5.03	4.50	5.18	1.23	4.22
	2	2.84	3.43	3.65	6.14	2.07	2.09	5.02	5.00	5.22	1.44	4.22
	3	2.16	2.09	2.82	5.44	1.63	1.51	4.15	4.35	4.41	0.33	3.47
	4	2.48	0.77	2.06	4.69	1.46	1.47	3.53	4.35	3.87	− 1.23	3.39
	5	1.95	2.17	2.95	5.24	1.52	1.53	4.20	4.07	4.56	0.59	3.83
July	1	3.20	2.28	1.72	5.03	2.32	1.49	3.12	4.38	4.04	0.30	3.87
	2	2.86	2.73	2.21	4.56	1.78	1.75	3.59	4.21	4.18	0.29	3.56
	3	2.53	2.80	3.83	5.97	2.19	2.15	4.95	5.14	5.45	1.71	4.33
	4	2.84	3.15	4.05	6.21	2.26	2.17	5.14	5.37	5.71	2.09	4.24
	5	3.21	2.13	1.71	5.00	2.32	1.52	3.11	4.32	4.10	0.27	3.68
August	1	2.85	3.81	3.11	5.76	2.16	1.94	4.38	5.11	5.18	1.32	4.05
	2	3.18	3.11	1.76	4.52	2.13	1.17	2.73	4.06	4.18	1.31	3.41
	3	2.81	4.10	3.92	6.30	2.24	2.14	5.11	5.31	5.67	1.89	4.34
	4	2.63	3.86	3.77	6.01	2.16	2.09	4.97	4.58	5.82	1.62	4.33
	5	2.73	3.92	3.85	6.02	2.12	2.18	5.02	4.70	5.90	1.55	4.61
September	1	3.48	3.41	2.66	5.43	2.13	2.25	3.70	4.72	5.03	0.61	3.86
	2	3.35	3.30	2.10	5.17	3.02	1.57	3.20	4.31	5.47	0.48	3.85
	3	2.66	3.18	2.49	5.09	2.57	1.72	3.79	4.47	5.20	0.84	4.72
	4	2.61	3.81	4.00	6.09	2.34	2.21	5.13	4.58	6.02	1.70	4.45
	5	2.61	3.87	3.75	6.10	2.16	2.12	5.05	4.53	5.83	1.58	4.42
Background value [mg/kg] (Lis and Pasieczna 1998),^*^(Kabata-Pendias and Mukherjee 2007)		75	1	3	29	5	13	0.5	16	7	0.5*	2
*I*_geo_ class		III	IV	IV	VI	III	II	V	V	VI	II	V

#### Pollution Load Index

The obtained results of *I*_geo_ calculations (Table 6) indicated the necessity to determine a PLI for four elements: Zn, Cd, Pb, and Cr. Pollution load index (Tomlinson et al. 1980) was used to determine the level of contamination of the bottom sediments compared to levels of background concentrations (Lis and Pasieczna 1998). Regarding the work of Liu et al. (2013), four levels of pollution have been defined: no pollution (PLI < 1), medium pollution (1 < PLI < 2), heavy contamination (2 < PLI < 3), and extremely strong pollution (PLI > 3).

According to the PLI classification (Tomlinson et al. 1980; Liu et al. 2013), the obtained results indicate that the bottom sediments of the Kozłowa Góra Reservoir were extremely heavily contaminated (PLI > 3). Calculated PLI for particular months of sampling (for Zn, Cd, Pb, and Cr: May-64.36, June-52.03, July-49.36, August-61.25, September-62.14) indicates that the largest Zn, Cd, Pb, and Cr contamination of the bottom sediments occurred in May and September. However, taking into spatial variability of PLI for Zn, Cd, Pb, and Cr is accordingly: first sampling point–54.63, second sampling point–48.99, third sampling point–64.92, fourth sampling point–64.81; fifth sampling point–55.88). The bottom sediment PLI also allows to conclude that the largest metal accumulation in the bottom sediments of the reservoir occurs in the third and fourth sampling point, i.e., in the middle of the Kozłowa Góra Reservoir. This is probably due to the morphometry of the tank, in which contaminants travel in the test transect, towards the water flow of the reservoir, to be deposited at the third and fourth bottom sediment sampling point.

## Conclusion

The examined methodology enabled obtaining reliable results despite low concentrations of metal(loid)s. The Dionex IonPac AS7 column was used for the simultaneous determination of five arsenic and antimony species [As(III), As(V), Sb(III), Sb(V), SbMe_3_]. Despite the developed and validated methodology, no methyl derivatives of antimony were found in the water or sediments of the Kozłowa Góra Reservoir, and the dominant form was Sb(V).

The variability of the physicochemical parameters studied in the water samples collected at four sampling points over a period of 5 months was observed. The obtained results indicate the influence of physicochemical conditions on changes in the antimony ionic form concentration. Our research has shown that the easy-leached bottom sediment fraction of the Kozłowa Góra Reservoir contained in most cases more As(V) and Sb(V). But in many cases, Sb(V) concentration was equal as Sb(III), which can be released into the pelagic zone under favorable conditions. Even though As(V) and Sb(V) prevail in the reservoir bottom sediments, they can be transformed into As(III) and Sb(III) as a result of drastic changes in pH or redox potential. The Kozłowa Góra bottom sediments are heavily polluted, primarily with heavy metals, such as Pb, Zn, Cd, and to a lesser extent, with As, Cu, and Ni. The values of PLI and *I*_geo_ coefficients determined for five sampling points confirmed that the Kozłowa Góra Reservoir ecosystem is polluted with heavy metals to a significant degree. The highest concentrations of the heavy metals tested were recorded in the middle of the tank (third and fourth sampling point). In the bottom sediments of the Reservoir, there is a small spatial variability of metal and metalloids concentrations. The migration of metals along the reservoir transect is closely related to its morphometry.
